# A Research Agenda to Better Understand the Human Dimensions of Energy Transitions

**DOI:** 10.3389/fpsyg.2021.672776

**Published:** 2021-06-25

**Authors:** Linda Steg, Goda Perlaviciute, Benjamin K. Sovacool, Marino Bonaiuto, Andreas Diekmann, Massimo Filippini, Frank Hindriks, Cecilia Jacobbson Bergstad, Ellen Matthies, Simon Matti, Machiel Mulder, Andreas Nilsson, Sabina Pahl, Martha Roggenkamp, Geertje Schuitema, Paul C. Stern, Massimo Tavoni, John Thøgersen, Edwin Woerdman

**Affiliations:** ^1^Faculty of Social and Behavioural Sciences, University of Groningen, Groningen, Netherlands; ^2^Science Policy Research Unit, University of Sussex, Brighton, United Kingdom; ^3^Faculty of Medicine and Psychology, Sapienza University of Rome, Rome, Italy; ^4^Department of Humanities, Social and Political Sciences, Eidgenössische Technische Hochschule Zürich, Zürich, Switzerland; ^5^Department of Management, Technology, and Economics, Eidgenössische Technische Hochschule Zürich, Zürich, Switzerland; ^6^Faculty of Philosophy, University of Groningen, Groningen, Netherlands; ^7^Department of Psychology, University of Gothenburg, Gothenburg, Sweden; ^8^Institute of Psychology, Otto von Guericke Universität Magdeburg, Magdeburg, Germany; ^9^Department of Business Administration, Technology and Social Sciences, Luleå University of Technology, Luleå, Sweden; ^10^Faculty of Economics and Business, University of Groningen, Groningen, Netherlands; ^11^School of Psychology, University of Plymouth, Plymouth, United Kingdom; ^12^Faculty of Law, University of Groningen, Groningen, Netherlands; ^13^College of Business, University College Dublin, Dublin, Ireland; ^14^Social and Environmental Research Institute, Greenfield, MA, United States; ^15^Department of Management, Economics and Industrial Engineering, Politecnico di Milano, Milan, Italy; ^16^Department of Management, Aarhus University, Aarhus, Denmark

**Keywords:** energy transition, sustainable energy behaviour, social sciences, humanities, research agenda, behaviour change, public support

## Abstract

The Social Sciences and Humanities (SSH) have a key role to play in understanding which factors and policies would motivate, encourage and enable different actors to adopt a wide range of sustainable energy behaviours and support the required system changes and policies. The SSH can provide critical insights into how consumers could be empowered to consistently engage in sustainable energy behaviour, support and adopt new technologies, and support policies and changes in energy systems. Furthermore, they can increase our understanding of how organisations such as private and public institutions, and groups and associations of people can play a key role in the sustainable energy transition. We identify key questions to be addressed that have been identified by the Platform for Energy Research in the Socio-economic Nexus (PERSON, see person.eu), including SSH scholars who have been studying energy issues for many years. We identify three main research themes. The first research theme involves understanding which factors encourage different actors to engage in sustainable energy behaviour. The second research theme focuses on understanding which interventions can be effective in encouraging sustainable energy behaviour of different actors, and which factors enhance their effects. The third research theme concerns understanding which factors affect public and policy support for energy policy and changes in energy systems, and how important public concerns can best be addressed as to reduce or prevent resistance.

## Introduction

Many states have set ambitious goals to decarbonise the energy system (Mundaca et al., 2019; Black et al., 2021). To achieve these goals, changes in technology *and* human behaviour are critical (IPCC, 2018; IEA, 2020, 2021). Sustainable energy systems require an active engagement of a wide range of actors including individual consumers, households, companies and organisations. Specifically, energy systems that rely on variable renewable energy sources will be more efficient and sustainable when energy demand is reduced and when energy demand better matches the supply of low carbon energy sources such as renewables. Energy users can support and engage in a wide range of sustainable energy behaviours that would promote sustainable energy systems and reduce greenhouse gas (GHG) emissions, including the adoption and use of renewable energy sources and low carbon innovations, increasing the energy efficiency of buildings, adoption of energy efficient appliances and vehicles, and changing behaviour associated with energy use in buildings and for transport to reduce fossil energy use (IPCC, 2018). Next, successful implementation of changes in energy systems and energy and climate policy require public support and political will (IPCC, 2018).

The need for Social Sciences and Humanities (SSH) research on the transition to sustainable energy systems is widely advocated, and is seen as pivotal to address the urgent challenges to achieve a successful transition (ISSC UNESCO, 2013; Hackmann et al., 2014; Sovacool, 2014; Weaver et al., 2014; Clayton et al., 2015; IPCC, 2018). Indeed, the SSH have a key role to play in understanding which factors and policies would motivate, encourage and enable different actors to adopt a wide range of sustainable energy behaviours. Moreover, SSH research can reveal which factors affect support for policies and system changes, and how to achieve sustainable energy systems that secure or even improve individual quality of life. Specifically, the SSH can provide critical insights into how consumers could be empowered to consistently engage in sustainable energy behaviour, and to support policies, technologies, and changes in energy systems. Furthermore, they can increase our understanding of how to motivate and enable organisations such as private and public institutions, and groups and associations of people to achieve a sustainable energy transition. Moreover, SSH research is needed to understand the impact of the sustainable energy transition on individual quality of life and the sustainable development goals.

The SSH has provided some important insights into the human dimensions of sustainable energy transitions, including factors influencing sustainable energy behaviour, and the effects and support for sustainable energy technologies and policies (e.g., Steg et al., 2015; Stern et al., 2016; Creutzig et al., 2018; Bouman and Steg, 2019; Ejelov and Nilsson, 2020; Nielsen et al., 2021). Yet, the SSH literature on the sustainable energy transition is fragmented and important gaps still need to be addressed. Below, we identify key gaps and questions to be addressed that have been identified by the Platform for Energy Research in the Socio-economic Nexus (PERSON, see person.eu), including SSH scholars who have been studying energy issues for many years. We identify three main research themes. The first research theme involves understanding which factors encourage different actors to engage in sustainable energy behaviour. The second research theme focuses on understanding which interventions can be effective in encouraging sustainable energy behaviour of different actors, and which factors enhance their effects. The third research theme concerns understanding which factors affect public and policy support for energy policy and changes in energy systems, and how important public concerns can best be addressed as to reduce or prevent resistance and to enhance positive outcomes for society at large.

## Research Themes

### Understanding (Un)sustainable Energy Behaviour

#### Contextual Factors Influencing Sustainable Energy Behaviour

Policy and system changes will be more effective if they consider and target key factors that promote or inhibit sustainable energy behaviour of different actors. The attractiveness and feasibility of sustainable energy behaviour depends on the context in which such decisions are being made, which is shaped by economic, political, institutional, legal, technological, social and cultural factors (Steg and Vlek, 2009; IPCC, 2018). It is important to understand to what extent different contextual factors inhibit sustainable energy behaviours, and which contextual factors would enable and empower different actors to act sustainably. Various actors, including governments, industry, business organisations, and civil society, can promote such contextual changes and enable and empower people to contribute to the sustainable energy transition. Therefore, it is critical to understand which factors affect the likelihood that governments, industry, and civil society will take action to change key contextual factors.

Furthermore, we need to understand path dependencies and the inertia of energy systems against (sustainable) changes, and how to prevent or overcome “lock-in” effects that can inhibit a sustainable energy transition (Kanger et al., 2019; Kotilainen et al., 2019). This requires an interdisciplinary and multilevel perspective on socio-technical innovations and technologies, as to understand which policies (e.g., laws, regulations, pricing schemes), governance, and motivational factors (such as values, attitudes, norms, and preferences) can encourage the adoption and use of technologies and innovations embedded in sustainable systems (Thøgersen, 2018).

#### Factors Influencing High-Impact Behaviour

Various studies have been conducted to better understand sustainable energy behaviour of individuals and households, mostly producing knowledge on factors influencing everyday energy-related behaviours and practises, such as switching off lights, recycling, showering time, and car use (Nielsen et al., 2021). Future research could examine whether the same factors influence behaviours that have a high potential for reducing GHG emissions. Notably, drivers and impediments of household energy investments that reduce fossil energy use have been understudied, including investments in sustainable energy production (e.g., solar panels; Wolske et al., 2017), building renovation and insulation, low carbon innovations (e.g., heat pumps), energy management systems (to reduce overall energy demand and to better match energy demand to the available supply of renewables; Murtagh et al., 2014; Toft et al., 2014; Noppers et al., 2019), energy storage facilities (e.g., batteries; Agnew and Dargusch, 2017; Thomas et al., 2019), and energy efficient appliances (see Kastner and Stern, 2015, for a review). This is particularly important as many of such investment behaviours are associated with a relatively high GHG emissions reduction potential and may thus be critical to meet ambitious climate targets (Dietz et al., 2009; Stern, 2020).

Specifically, we need to identify which cognitive, motivational, social, cultural, physical and institutional factors influence the adoption of different sustainable innovations and technologies by individuals, households and organisations (Kastner and Stern, 2015). In doing so, it is important to understand whether different factors affect adoption likelihood of earlier versus late adopters (Noppers et al., 2015; Toft and Thøgersen, 2015). Also, we need a better understanding of the ways in which people and sustainable energy technology interact (Midden et al., 2007), which would provide critical insights in how to promote the adoption and optimal use of sustainable energy technology.

Next, it is important to understand which factors affect housing and location decisions (Stern et al., 2016), as choices for, among others, smaller living space, co-housing, working close to home and/or telecommunicating could substantially contribute to reducing total GHG emissions (Grübler et al., 2018; Ivanova et al., 2020). Relevant questions include: what motivates people to move to smaller houses or low-energy houses, and which factors influence the likelihood that they choose locations for living, work, shopping and leisure that would reduce their (motorised) travel needs? Similarly, which factors affect location decisions of companies and industry that would reduce transport distances?

We do not only need to understand which factors affect direct energy use, but also study indirect or embedded energy use by individuals and households, that is, the amount of energy used to produce and transport products and services, which constitutes a substantial proportion of the total energy used by individuals and households (Vringer and Blok, 1995; Ivanova et al., 2020). Again, it would be particularly important to study behaviours associated with high levels of GHG emissions, such as meat and dairy consumption (IPCC, 2018; Ivanova et al., 2020). Relatedly, it is important to understand which factors affect behaviour in line with a circular economy that are associated with lower GHG emissions, including reducing waste (such as food waste), recycling, repairing, reusing, and sharing products (Camacho-Otero et al., 2018).

#### Factors Influencing the Likelihood of Broader Lifestyle Changes

Research on how different types of actions, including everyday energy-use behaviour, investments, and policy support, are linked and how broader sustainable lifestyle changes can be achieved is still emerging. A key question in this respect is whether and to what extent rebound and spillover effects may occur (Truelove et al., 2014). For example, how can we prevent that energy saving actions lead to additional energy demand (e.g., increased driving after switching to a more fuel-efficient car, or using financial savings from energy conservation measures to fly to holiday destinations; IPCC, 2018)? Will engagement in actions that reduce fossil energy use provide a licence to refrain from other energy-saving actions (i.e., “negative spillover”), and if so, under which conditions is this most likely to be the case? More importantly, which factors promote “positive spillover,” in which case actors would consistently engage in sustainable energy behaviour, over and again, in many different situations, which is needed to achieve a truly sustainable energy transition (Nash et al., 2019)?

#### The Effects of General Motivational Factors on Sustainable Energy Behaviours

Given the large diversity of actions needed to realise and accelerate sustainable energy transition, it is important to better understand the ways in which general factors such as values, self-identities, social identities, and cultural factors could affect many different behaviours over and again, and under which conditions they are more likely to do so (Sovacool and Griffiths, 2020; Bouman et al., 2021). Research suggests that values that reflect concern with others (i.e., altruistic values), and particularly values that reflect concern with nature and the environment (i.e., biospheric values) are most likely to predict consistent sustainable energy behaviour and energy policy support (Steg, 2016).

Yet, people do not seem to consistently act upon their altruistic and biospheric values, as reflected in a so-called value-behaviour gap (Steg, 2016). Hence, understanding which factors cause the value-behaviour gap, and which factors induce actors to act more consistently upon their altruistic and biospheric values may improve the likelihood of influencing many behaviours in different situations through types of intervention rather than merely seeking to influence single behaviours one at a time.

Moreover, we need to understand how values and norms are formed and transformed in different societies and groups (Bardi and Goodwin, 2011). Individuals are products of social, political, cultural and ideological contexts that may affect their sustainable energy behaviour in important ways. It is important to understand the impacts of cultural variability, and to study how social and cultural factors affect behaviour, vis-à-vis individual factors. Individual beliefs and behaviour may be affected by different cultural layers, ranging from the global, national, regional, and group level, including specific groups like families and companies (Fielding and Hornsey, 2016; Jans et al., 2018).

#### Judgmental Biases

More research is needed into biases that may inhibit adequate judgements and optimal decisions related to energy behaviour of various actors (IPCC, 2018; Hahnel et al., 2020). Important questions to be addressed are which judgmental biases inhibit sustainable energy behaviour, and which processes underlie such decision-making. For example, it is important to understand which factors encourage investments in sustainable energy solutions with high upfront financial costs while their financial benefits are only apparent in the long term. Further, people may misjudge the GHG emissions and possible energy savings of their behaviours (Attari et al., 2010; Lesic et al., 2019), and it is important to understand what is needed for people to make more accurate assessments.

Deviations form ideally “rational” decision-making may not only be prevalent among individuals and households, but also among firms and politicians. We need to understand which factors and mechanisms underlie this supposed irrationality in policy making, as to enhance the overall effects and cost-efficiency of energy policies.

#### Factors Influencing Sustainable Behaviour of Organisations, Firms, Industry, and Governments

We not only need a better understanding of factors influencing sustainable energy use of individuals and households, but also of organisations, firms, industry and governments. Notably, organisations, including commercial, governmental and non-profits, account for a major share of total energy use, but little is known about factors influencing their sustainable actions (Huffman and Klein, 2013; Stern et al., 2016; Wells et al., 2018). Also, choices of organisations, firms and industry can have a major impact on the attractiveness and feasibility of individuals' sustainable energy behaviours (Stern and Dietz, 2020). We need to understand which factors affect energy use in the operational practises of organisations as well as which factors influence the likelihood that organisations facilitate fossil energy savings among their clients by designing and marketing products associated with lower carbon emissions. A relevant question here is to what extent, how, and under which conditions the mission and strategies of an organisation, such as the extent to which organisations prioritise profit generation vs. corporate environmental sustainability, affects their sustainable energy use and CO_2_ emissions (Ruepert et al., 2017). Further buttressing this work could be explorations of factors influencing behaviour of people in different roles (e.g., in organisations, governments, community organisations, and investors).

One promising theme to be explored is the role of intermediaries, those who act as brokers or agents between different groups of people or institutions (Kivimaa and Martiskainen, 2018; Sovacool et al., 2020). For example, household carbon footprints in Northern Europe shifted meaningfully over time following a household lifecycle, as a function of major decisions (such as purchasing a car or home) or major life events (such as having children or getting divorced; Dubois et al., 2019). Hence, particular intermediaries who affect choices during such life events, such as estate agents for homes, plumbers, retirement planners, and car dealerships, could shape preferences and choices. This is particularly important as intermediaries seem deceptive and dismissive toward some lower-carbon options, such as battery electric vehicles (Zarazua de Rubens et al., 2018). More research is needed to understand the conditions under which intermediaries can promote or inhibit sustainable energy behaviour in different domains.

#### Factors Influencing Sustainable Energy Behaviour in Developing and Emerging Countries

Finally, a better understanding of factors influencing (un)sustainable energy behaviour in developing and emerging countries is critical, as this will provide crucial insights in how to reach global GHG emission targets (IPCC, 2018). It is particularly important to understand to what extent similar factors and processes affect sustainable energy behaviour across the world, and how to reduce energy poverty without increasing carbon emissions. Also, research is needed on possible conflicts of interest between different countries that may inhibit a sustainable energy transition, to develop solutions in which costs and benefits are distributed across countries in a fair way. Certain solutions, such as offshore wind energy developments, have implications for multiple countries that raises the question what new (international) governance structures and legal instruments may be required (IPCC, 2018).

### Interventions to Promote Sustainable Energy Behaviour

#### Effects of Different Types of Interventions to Promote Sustainable Energy Behaviour

It seems unlikely that actors will engage in a wide range of sustainable energy behaviour without additional inducements, particularly since various contextual factors that cannot be controlled by individuals can inhibit or demotivate sustainable energy behaviour (Stern, 2020). Governments at all levels could implement policies including laws, regulations, standards and rules; pricing policies; changes in the infrastructure; and social incentives that facilitate the transformation to sustainable energy systems (IPCC, 2018).

Similarly, industry, business organisations and civil society can take various actions that facilitate and promote sustainable energy choices, and remove important barriers for change. We need to better understand the psychological and behavioural effects of contextual changes that aim to make sustainable energy behaviour more attractive or feasible (Steg and Vlek, 2009; Stern, 2020). Specifically, we need to increase our understanding of the conditions under which different contextual changes are most effective, how negative side effects can be prevented, and the role of governments and other actors in creating and implementing different changes and incentives for various actors.

Various studies have evaluated the effects of regulatory and non-regulatory interventions aimed at promoting sustainable energy behaviour. Most of these studies examined the effects of informational strategies and economic incentives on individual or household energy use behaviour (for reviews see Abrahamse et al., 2005; Dietz et al., 2009; Abrahamse and Steg, 2013; Bolderdijk and Steg, 2015; Maki et al., 2016; Mundaca et al., 2019). These studies provided important insights into the extent to which and when different interventions, alone or in combination, can be effective to promote sustainable energy behaviour. Pricing policy proved to be effective to encourage sustainable energy behaviour (Maki et al., 2016; Wolske and Stern, 2018; Bayer and Aklin, 2020), but may have negative side effects as well, such as decreasing individuals' intrinsic motivation to act sustainably, which can inhibit consistent sustainable energy behaviour (Bolderdijk and Steg, 2015). Yet, generally, the processes through which different types of strategies encourage sustainable energy behaviour have hardly been studied, so little is known about why and under which conditions different policies and strategies are most effective. Similarly, knowledge is quite incomplete on how insights from SSH can enhance the effects of incentives (Stern et al., 2010). These are important questions for future research, as such insight are critical to optimise policy instruments. Moreover, some other important questions remain.

#### How to Enhance the Impact of Bottom-Up Initiatives

More research is needed into the conditions under which social influence approaches are most effective in promoting sustainable energy behaviour. A meta-analysis suggests that social influence strategies may be particularly effective when they involve social interaction (Abrahamse and Steg, 2013). Promising developments in this respect are local initiatives and self-organisation to promote sustainable energy behaviour. However, it is unclear to what extent and under which conditions such bottom-up initiatives are effective in encouraging sustainable energy behaviour, what motivates people to take initiative or to join a bottom-up sustainable energy initiative, and how collectives that are formed from the bottom-up function and sustain (Sloot et al., 2018, 2019). Also, it is as yet not clear whether and when such initiatives are more effective than top-down policies, and how synergies with top-down policies can be optimised. Moreover, research is needed to examine which policies, institutional changes, governance structures, and legal regimes would support changes from the bottom-up.

#### Ways to Strengthen People's Intrinsic Motivation to Act Sustainably

A better understanding is needed of ways to strengthen individuals' intrinsic motivation to engage in sustainable energy behaviour, as this may be an important source for consistent sustainable behaviour. Notably, people are intrinsically motivated to do the right thing and to protect the environment, and doing so elicits positive feelings and enhances well-being (Steg, 2016; Johnson Zawadzki et al., 2020; Zawadzki et al., 2020). We need to better understand how such intrinsic motivation can be fostered and strengthened. Specifically, we need to understand which factors affect the relative strength of biospheric and altruistic values, as acting pro-environmentally is likely to be particularly intrinsically rewarding to those who strongly endorse these values (Venhoeven et al., 2020). Although some studies revealed that value strength can change over time, little is known about how such changes can be achieved, and under which conditions people are likely to reconsider the prioritisation of their values (Bardi and Goodwin, 2011). Moreover, research should improve our understanding of which contextual factors enhance actors' intrinsic motivation to protect the environment, and under which conditions this would promote sustainable energy behaviours. There is initial evidence to suggest that contextual factors can foster intrinsic motivation to engage in sustainable energy behaviour (Steg et al., 2014; Steg, 2016), but more research is needed to test this account.

#### Identify Which Costs and Benefits of Sustainable Energy Behaviour Can Best Be Emphasised

Research should improve our understanding of which costs and benefits can best be emphasised to promote sustainable energy behaviour. Notably, energy efficiency measures such as home insulation can yield co-benefits such as improved indoor air quality. Similarly, electric vehicles can reduce noise pollution, while many energy saving behaviours would also imply cost savings. We need to better understand to what extent and when emphasising such co-benefits can be effective in promoting sustainable energy behaviour, and when emphasising co-benefits would rather be counter effective (Asensio and Delmas, 2015; Schwartz et al., 2015).

#### Promote Active Engagement in Sustainable Energy Systems

We need to better understand which strategies can encourage individual engagement in sustainable energy systems, which is critical to enhance the efficiency and sustainability of such systems. Notably, smart grids that increasingly rely on renewable energy sources provide the opportunity to inform, involve, and engage consumers by providing them information about their energy use and, if appropriate, their energy production, as well about the consequences thereof, including financial costs and carbon emissions. We need to comprehend how to best design and convey relevant information and feedback to motivate consumers to reduce their energy use and to match their energy demand to the available (renewable) energy supply to increase the efficiency and sustainability of smart grids (Toft and Thøgersen, 2015; Batalla-Bejerano et al., 2020). More generally, we need to examine how to best design energy-related information to increase energy literacy (Blasch et al., 2021), to enhance the understanding and persuasiveness of such information among different groups. Next to written information, images and ambient feedback needs to be tested (Goodhew et al., 2015). Also, appropriate legal frameworks need to be developed to support the exchange of private energy information and enhance trust in information sharing systems that may be critical for effective functioning of smart grids.

#### Synergies and Trade-Offs Between Policies, and Impacts of Energy Policies on the UN's Sustainable Development Goals and Individuals' Quality of Life

It is critically important to reveal possible synergies and trade-offs between different energy policy instruments, and how current policies and governance structures may facilitate or inhibit sustainable energy behaviour of various actors. This will provide important insights into effective policy mixes to encourage sustainable energy behaviours. Lock-in effects of policy and institutional and regulatory frameworks in place are a related issue, which may give rise to policy conflicts that can inhibit the transition to sustainable energy systems. More generally, we need to understand the interactions among energy policies and between energy and other policies across sectors and levels, and examine how policy coherence can be enhanced by strengthening policy synergies and preventing policy conflicts. This will reveal to what extent energy and climate policies have positive vs. negative effects on other policy goals, including the United Nations Sustainable Development Goals (see https://sdgs.un.org/goals), and human health and well-being in general (IPCC, 2018). Also, it will improve our understanding of whether and how environmental policy integration can be achieved, following a multisector and multi-level governance approach, thereby negotiating interests of policy actors at different levels.

#### How to Promote Sustainable Actions by Governments, Firms, and Organisations

As indicated above, we not only need a better understanding of how to change behaviour of individuals and households, but also how to change actions by governments, firms and organisations. How can successful business models for sustainable energy innovations and energy systems be created, and which governmental policies are needed to secure profitability of innovative sustainable energy businesses? How might programmes focusing more on missions and challenges, rather than disciplines, catalyse innovation more effectively (Sanchez and Sivaram, 2017)? Here, questions like confidence in long-term commitments by the State or other actors to govern toward sustainable energy systems could be key. A related challenge is the financing of the sustainable energy transition. Who is willing to make the required investments, why, under which conditions, and at what price? Are institutional investors willing to participate, do we need public funding, and what is the scope and potential for crowd-sourcing?

#### Differences and Similarities of Effects of Sustainable Energy Policies Across Regions and Cultures

Apprehending relevant cross-cultural and regional similarities and differences is important to develop and implement effective sustainable energy policies across the world. It is particularly critical to get a better understanding of which policies are effective in promoting sustainable energy behaviour in developing countries that also benefit other SDGs and help eradicate poverty, which have been understudied. This could help these countries in adopting best practises, and to accelerate the sustainable energy transition worldwide. At the same time, successful experiences in developing countries can inform sustainable energy transitions in the developed world, such as how to establish and manage decentralised energy systems (Giner-Reichl, 2015; Baptista, 2018).

### Public and Political Support for Sustainable Energy Systems and Policies

Many potential effective energy policies and innovations are not implemented because of (perceived) lack of public and political support. Therefore, it is crucial to better understand which factors affect support for energy policies, energy system changes, energy infrastructure and innovations, and how to address public concerns so that broader positive societal outcomes can be achieved. As yet, studies on public support are fragmented, and processes influencing public support are not well-understood.

Research has shown that public support is higher when a policy is expected to have less negative and more positive effects, when costs and benefits are distributed in a fair way, and when the decision-making process is seen as fair (Perlaviciute and Steg, 2014; Drews and Van den Bergh, 2016). Yet, more research is needed to understand which costs and benefits are important for different actors or groups, and which fairness principles drive public support. Furthermore, we need to understand how public concerns about energy policies and innovations can best be addressed, for example by providing information on possible risks, costs and benefits, by changing characteristics of policies and innovations, by changing the plans altogether, and by changing the ways of decision making and implementation of policies.

#### Factors Influencing the Perceived Fairness of Policies and Public Support

Addressing pressing issues of equity, justice, vulnerability and fairness would humanise aspects of energy consumption or transitions (Lamb et al., 2020; Sovacool, 2021). Yet, little is known about which factors affect perceived legitimacy and fairness of policies and how this in turn affects the support for energy policies and system changes and individuals' quality of life. Philosophical analyses of climate justice in general and energy fairness in particular provide frameworks for answering such questions (Caney, 2011; Jones et al., 2015).

People perceive decision processes as more fair when they think that their values, interests, and concerns have been considered, when they feel they can participate and have a “voice” in decision-making and can influence decisions, and when processes focus on respect, openness, and honesty (Bidwell, 2016; Evensen et al., 2018). In contrast, people tend to resist decisions when they feel that they have been involved too little and too late in the decision-making (Gross, 2007; Walker and Baxter, 2017; Liu et al., 2019). Yet, little is known about when and how people wish to be involved in the decision-making and under which conditions this will actually enhance public support (Perlaviciute and Squintani, 2020). We need to grasp factors influencing the effects of participative and interactive policy making, and under which conditions this leads to more democratic, substantively better (e.g., by integrating local knowledge), and more legitimate and acceptable policies and decisions (Pidgeon, 2021). Moreover, research is needed into how participative processes can best be organised to motivate participation and effectively incorporate different values, interests, and concerns (e.g., *via* deliberative processes; Dietz, 2013; Pidgeon et al., 2014) and what type of involvement enhances public support (e.g., representation vs. direct participation; Bernauer and Gampfer, 2013; Bernauer et al., 2016).

#### Public Support for Changes in Choice Context and Novel Technologies

Also, research is needed into public support for energy policies aimed to improve the context in which energy choices are made, including new energy standards, energy labelling, energy-related taxes and subsidies, tradable emission allowances, and nudges (e.g., default options that can promote adoption of green energy tariffs; Liebe et al., 2018, 2021). Next, acceptability of technologies that aim to increase the efficiency and sustainability of smart grids need to be studied (Toft and Thøgersen, 2015). In particular, more knowledge is needed on how to effectively address privacy concerns when data on energy production and use is likely to be shared (Bolderdijk et al., 2013). In addition, more research is needed into the acceptability of smart grid technologies that aim to improve the matching of production and use of energy, as to increase the efficiency of the system (Murtagh et al., 2014; Toft et al., 2014). For example, under which conditions are people likely to accept automated remote-control systems, how can we meet people's need to feel in control over the relevant systems, and which factors affect trust in the relevant systems? Which legal frameworks are needed to support such new technologies and to secure privacy protection?

It is important to better understand which factors influence public support for siting and design of installations, as public opposition to renewable energy developments and related grid projects can be a major barrier to a sustainable energy transition (Devine-Wright, 2005; Thøgersen and Noblet, 2012; Temper et al., 2020). Among others, we need to better understand how to improve decision-making, communication and procedural issues (on a local and national level) related to siting and design of installations, and how this can enhance support for decisions.

#### Factors Influencing Political Support and Interactions Between Public and Political Support

Besides public support, it is important to consider factors influencing political support for sustainable energy policies and a sustainable energy transition. It is important to understand which factors and processes underlie political decision-making that may slow down or threaten a sustainable energy transition. Also, we need to appreciate what motivates politicians and decision-makers to implement policy to promote sustainable energy behaviours (Rickards et al., 2014).

We need to apprehend how public support and political support interact. For example, politicians may not want to implement policies that are likely to evoke public resistance. It is important to understand how politicians form their perceptions of public opinion and how much these perceptions resemble what the public actually thinks. Likewise, it is important to appreciate whether, how, and under which conditions political support may affect public support, for example because the public appreciates political leadership, or because of enhanced trust in the leadership (Dietz et al., 2015; Zawadzki et al., 2020). Moreover, it is important to understand how interest group lobbying affects political and public support (Oreskes and Conway, 2010).

#### Factors Influencing Social Support for Alternative Models of Prosperity and Sustainable Growth

The continuing growth in global consumption in both material and economic terms contributes to environmental degradation, but apparently it contributes little to well-being in the industrialised countries. Research has identified a complex and interacting set of causes of consumption growth (Thøgersen, 2014). Understanding how people may live a satisfactory life with a lower environmental impact has major practical and policy implications for the environment, economic development, and energy security issues (Venhoeven et al., 2013). More insight is needed into alternative models of prosperity and sustainable growth, and the extent to which these models are acceptable to different actors (Jackson, 2021). Research is needed to develop alternative economic models on the link between energy consumption and well-being (Van den Bergh, 2018).

## Harnessing Research Insights for a Low-Carbon Future

To successfully implement ambitious long-term energy transition strategies, better knowledge on the social and behavioural dimension of energy systems is urgently needed. Drawing from this review, Table 1 summarises the key overarching questions for each of our three core research themes. As Table 1 implies, interdisciplinary collaboration across SSH, and between SSH and other disciplines, is key to understand the complex nature of the human dimension of energy problems, and to offer policymakers a more complete understanding of ways to accelerate the sustainable energy transition (Clayton et al., 2015). Notably, interdisciplinary research is needed as many different players, markets, institutions, and technologies influence the likelihood of a sustainable energy system, the opportunities actors face, as well as costs and benefits of different possible sustainable energy solutions. Interdisciplinary projects are more likely to be successful when interdisciplinary collaborations already start in the problem formulations and planning stages of a project, so that collaborators agree on basic approaches, tasks, and programme coordination early on. Next, regular collaborative meetings are needed to discuss different ideas and approaches, and to secure initial divergent perspectives are timely converged (Schoot Uiterkamp and Vlek, 2007).

**Table 1:** Thematic research agenda on the human dimensions of sustainable energy transitions: summary of key questions.

**Understanding (un)sustainable energy behaviour**	**Interventions to promote sustainable energy behaviour**	**Public and political support for sustainable energy systems and policies**
• The effect of contextual factors on sustainable energy behaviour, and the role of organisations, industry and intermediaries in creating contexts that support sustainable energy behaviours • Factors influencing high-impact behaviour (e.g., giving up individual cars, reducing air travel, investment in renewable energy technology, insulating homes, housing and location decisions) • Factors influencing broader lifestyle changes and preventing rebound effects and negative spillovers • The effects of general motivational factors on sustainable energy behaviour • The effects of judgmental biases on sustainable energy behaviour • Factors influencing (un)sustainable behaviour of organisations, firms, and governments • Factors influencing sustainable energy behaviour in developing and emerging countries	• Which interventions (e.g., emphasising different costs and benefits, social influence approaches, community initiatives) are most effective in encouraging sustainable behaviour, why, and under which conditions • How to enhance the impact of bottom-up initiatives • Ways to strengthen people's intrinsic motivation to act sustainably • Identify which costs and benefits of sustainable energy behaviour can best be emphasised • How to promote active engagement in sustainable energy systems • Synergies and trade-offs between (energy) policies, and impacts of energy policies on Sustainable Development Goals and individuals' quality of life • How to promote sustainable actions by governments, firms and organisations • Differences and similarities in effects of sustainable energy policies across regions and cultures	• Factors influencing perceived fairness of energy policies, and how this in turn affects public support • Public support for changes in choice context and novel technologies • Understand which factors influence political support for change and interactions between public and political support • Factors influencing social support for alternative models of prosperity and sustainable growth

In sum, we maintain that SSH research on energy is key to improve and accelerate the decision-making and planning for sustainable energy transitions that are feasible, (cost-)efficient, supported by the public and policy makers, and that secure individuals' quality of life. Our proposed research agenda will increase our understanding of different actors' readiness to change their behaviours, the conditions under which such changes are most likely, and the extent to which different actors support policies and technological and system changes. Such insights are critical to design recommendations and guidelines on how to accelerate the sustainable energy transition across the world.

## Author Contributions

All authors listed have made a substantial, direct and intellectual contribution to the work, and approved it for publication.

## Conflict of Interest

The authors declare that the research was conducted in the absence of any commercial or financial relationships that could be construed as a potential conflict of interest.
